# Digital remote assessment of speech acoustics in cognitively unimpaired adults: feasibility, reliability and associations with amyloid pathology

**DOI:** 10.1186/s13195-024-01543-3

**Published:** 2024-08-01

**Authors:** Rosanne L. van den Berg, Casper de Boer, Marissa D. Zwan, Roos J. Jutten, Mariska van Liere, Marie-Christine A.B.J. van de Glind, Mark A. Dubbelman, Lisa Marie Schlüter, Argonde C. van Harten, Charlotte E. Teunissen, Elsmarieke van de Giessen, Frederik Barkhof, Lyduine E. Collij, Jessica Robin, William Simpson, John E Harrison, Wiesje M. van der Flier, Sietske A.M. Sikkes

**Affiliations:** 1 Alzheimer Center Amsterdam, Neurology, Amsterdam University Medical Center, De Boelelaan 1118, Amsterdam, 1081 HZ The Netherlands; 2 Amsterdam Neuroscience, Neurodegeneration, Amsterdam, The Netherlands; 3 Department of Clinical, Neuro and Developmental Psychology, Faculty of Movement and Behavioral Sciences, VU University, Amsterdam, The Netherlands; 4 Department of Neurology, Massachusetts General Hospital, Harvard Medical School, Boston, MA USA; 5 Alzheimer Center Groningen, Department of Neurology, Department of Neuropsychology and Department of Internal Medicine, University Medical Center Groningen, Groningen, The Netherlands; 6 Alzheimer Center Erasmus MC and Department of Neurology, Erasmus MC University Medical Center, Rotterdam, The Netherlands; 7 Center for Alzheimer Research and Treatment, Department of Neurology, Brigham and Women’s Hospital, Harvard Medical School, Boston, MA USA; 8 Neurochemistry Laboratory and Biobank, Department of Laboratory Medicine, Amsterdam Neuroscience, Amsterdam University Medical Center, Vrije Universiteit, Amsterdam, The Netherlands; 9 Department of Radiology & Nuclear Medicine, Amsterdam University Medical Center, Amsterdam, The Netherlands; 10 Amsterdam Neuroscience, Brain Imaging, Amsterdam, The Netherlands; 11 Queen Square Institute of Neurology and Centre for Medical Image Computing, University College London, London, UK; 12 Clinical Memory Research Unit, Department of Clinical Sciences, Faculty of Medicine, Lund University, Malmö, Lund, Sweden; 13 Cambridge Cognition, Bottisham, UK; 14 Scottish Brain Sciences, Edinburgh, UK; 15 Institute of Psychiatry, Psychology & Neuroscience, King’s College London, London, UK; 16 Department of Epidemiology and Biostatistics, Amsterdam Neuroscience, Amsterdam University Medical Center, Amsterdam, the Netherlands

**Keywords:** Alzheimer’s disease, Amyloid, Language, Speech acoustics, Remote assessment, Digital biomarker

## Abstract

**Background:**

Digital speech assessment has potential relevance in the earliest, preclinical stages of Alzheimer’s disease (AD). We evaluated the feasibility, test-retest reliability, and association with AD-related amyloid-beta (Aβ) pathology of speech acoustics measured over multiple assessments in a remote setting.

**Methods:**

Fifty cognitively unimpaired adults (Age 68 ± 6.2 years, 58% female, 46% Aβ-positive) completed remote, tablet-based speech assessments (i.e., picture description, journal-prompt storytelling, verbal fluency tasks) for five days. The testing paradigm was repeated after 2–3 weeks. Acoustic speech features were automatically extracted from the voice recordings, and mean scores were calculated over the 5-day period. We assessed feasibility by adherence rates and usability ratings on the System Usability Scale (SUS) questionnaire. Test-retest reliability was examined with intraclass correlation coefficients (ICCs). We investigated the associations between acoustic features and Aβ-pathology, using linear regression models, adjusted for age, sex and education.

**Results:**

The speech assessment was feasible, indicated by 91.6% adherence and usability scores of 86.0 ± 9.9. High reliability (ICC ≥ 0.75) was found across averaged speech samples. Aβ-positive individuals displayed a higher pause-to-word ratio in picture description (*B* = -0.05, *p* = 0.040) and journal-prompt storytelling (*B* = -0.07, *p* = 0.032) than Aβ-negative individuals, although this effect lost significance after correction for multiple testing.

**Conclusion:**

Our findings support the feasibility and reliability of multi-day remote assessment of speech acoustics in cognitively unimpaired individuals with and without Aβ-pathology, which lays the foundation for the use of speech biomarkers in the context of early AD.

**Supplementary Information:**

The online version contains supplementary material available at 10.1186/s13195-024-01543-3.

## Introduction

Speech production is one of the most distinctive traits of the human species, and an important tool for everyday communication [1]. It is a complex process, relying on multiple interacting cognitive functions [2, 3], thereby being susceptible to cognitive disruptions. Speech production on the level of acoustic speech characteristics is affected by many neurodegenerative diseases, including Alzheimer’s disease (AD) [4–7], a disease clinically characterized by a gradual decline in cognition, and biologically defined by amyloid-beta (Aβ) accumulation and neurofibrillary tau tangles [8]. These pathological processes begin in the preclinical AD stage, decades before cognitive symptoms are clinically objectified in the mild cognitive impairment (MCI) and dementia stages [9]. Detecting the earliest subtle signs of cognitive decline that may occur in the preclinical stage remains challenging.

To detect the earliest signs of cognitive decline, automatically extracted natural speech features are emerging as promising digital biomarkers of neurological diseases including AD [10]. For instance, in individuals with MCI due to AD, associations have previously been shown between Aβ-biomarkers and machine learning based acoustic scores, derived from multiple acoustic features. [11, 12] The current literature states that temporal acoustic speech features, such as the number and duration of pauses, are altered in AD [4, 6, 7, 13–15]. Acoustic features such as fundamental frequency, jitter (i.e., variation in frequencies) or shimmer (i.e., variation in amplitudes in decibels) of the voice have also been indicated to be related with clinically diagnosed AD in the MCI or dementia stage, although these voice characteristics have been studied less extensively and evidence is inconclusive. [13, 15, 16] To date, however, a knowledge gap remains on the association between individual acoustic features and Aβ pathology, specifically in individuals with preclinical AD. Generation of evidence on the relation between AD-specific pathology and acoustic speech changes is an important step towards using speech as a digital biomarker in the context of intervention studies. In addition, more insight is needed in whether such associations can be found in speech measured in an unsupervised, remote setting.

Major advantages of remote, at-home assessment of speech acoustics are that it enhances the ecological validity, potentially reduces patient burden, is highly scalable, and allows for high-frequent testing to provide a more reliable index of cognition [17]. Although speech characteristics have previously been shown to be measured with high test-retest reliability using tablet-based assessments [18, 19], more evidence on quality characteristics of remotely measured speech acoustics, such as its feasibility and test-retest reliability, is crucial to support the potential implementation of remotely measured speech acoustics as a digital biomarker. Test-retest reliability is considered an important measurement characteristic that should be attested to ensure a measurement is consistent for the same patient under the same conditions over a short period of time [20].

The present study aimed to investigate remotely measured acoustic characteristics of connected speech in cognitively unimpaired adults with and without Aβ pathology. Specifically, we examined (1) the feasibility of a remote multi-day tablet-based speech assessment to obtain speech recordings, (2) the test-retest reliability of remotely measured acoustic speech features over multiple assessments, and (3) the associations between remotely measured acoustic speech features and Aβ pathology.

## Methods

### Participants

We recruited 50 cognitively unimpaired participants between March and September 2022 from the memory clinic based Amsterdam Dementia Cohort (ADC [21, 22]) and embedded Subjective Cognitive Impairment Cohort (SCIENCe [23]), as well as from a population-based cohort, i.e., Amyloid Imaging to Prevent Alzheimer’s Disease Prognostic and Natural History Study (AMYPAD-PNHS [24, 25]). Participants included via ADC and SCIENCe were referred to a memory clinic, and diagnosed with subjective cognitive decline (SCD) in a multidisciplinary consensus meeting if clinical and cognitive examination fell within normal ranges and diagnostic criteria for MCI, dementia, or other psychiatric or neurological disorders were not fulfilled [23]. AMYPAD PNHS is a pan-European cohort of pre-dementia and mainly individuals with preclinical AD [24, 25]. We specifically selected cognitively unimpaired participants, based on Clinical Dementia Rating (CDR) = 0 [26] and Mini-Mental State Examination (MMSE) ≥ 26 [27].

Participants were eligible for inclusion if they were ≥ 50 years of age, had unimpaired cognition, were native speakers of Dutch, self-reported to have experience using smartphones or tablets, and had Aβ-biomarkers available that were obtained within 1.5 years of the speech assessments. Information on cognitive functioning and Aβ-biomarkers were derived from the cohort the participant was recruited from (see below). Exclusion criteria were the presence of other neurological or psychiatric diseases that may interfere with cognition, or self-reported major hearing or visual problems that limit testing procedures.

### Materials

#### Amyloid biomarkers

Aβ-biomarkers were previously obtained from either amyloid positron emission tomography-imaging (PET, *n* = 45) or cerebrospinal fluid (CSF, *n* = 5). For amyloid PET-scans [^18^F]flutemetamol (Vizamyl), [^18^F]florbetapir (Amyvid) or [^18^F]florbetaben (Neuraceq) tracers were used [23, 28, 29]. CSF was obtained by lumbar puncture, and Aβ_1−42_ concentrations in CSF were analyzed with electrochemiluminescence immunoassays (Roche Elecsys). Subsequently, dichotomized Aβ-status (positive/negative) was determined based on either visual inspection of amyloid PET-scans by an independent nuclear radiologist according to manufacturer guidelines, or local cutoffs in Aβ_1−42_ concentrations in CSF, where < 1000 pg/mL indicated positive Aβ-status [30, 31].

#### Speech assessment

The Winterlight Assessment application [18] (WLA app) was used to collect speech samples remotely from the participants’ home environment. The WLA has been explained in more details previously [18]. Speech tasks in the WLA app ranged from structured (i.e., verbal fluency) to unstructured (i.e., picture description, journaling) elicitation methods:

1. Picture description: Repetitive (5 sessions) and Alternating (5 sessions)

A line drawing depicting a particular scene was presented on the tablet screen, and participants were instructed to describe the scene, without a time limit. The line drawings resembled the widely used Cookie Theft Picture [32] in the amount of information content units and lexico-syntactic complexity [18]. In the speech assessment, two types of picture description tasks were included, with one of each type included per session: (A) repetitive picture description (henceforth: repetitive-PD), depicting a line drawing of a kitchen scene, kept constant across five sessions, and (B) alternating picture description (henceforth: alternating-PD), depicting a line drawing of a unique scene at each of the five sessions.

(2) Journaling (5 sessions)

An open-ended journaling prompt was displayed on the screen that aimed to elicit connected speech without a time limit. Journaling prompts included prompts designed by Winterlight Labs that were adjusted to Dutch cultural norms (i.e., “Could you describe what you like to do in your spare time, and elaborate on what this involves?” and “Could you elaborate on what you did yesterday?”), as well as questions that were adopted from a previous study [33], (i.e., “Could you tell what you do on a regular Sunday?”) or prompts that were partially based on speech tasks used in a previous study [34] (i.e., “Could you tell how you met one of your closest friends?” and “Could you elaborate on what you did during your last holiday?”).

(3) Verbal fluency: Phonemic (1 session) and Semantic (1 session)

In the verbal fluency tasks, participants were instructed to generate as many words starting with the letter D [35] (phonemic fluency), or as many animals [36] (semantic fluency), within a one-minute time limit.

Acoustic features were extracted from the speech recordings through automatic speech recognition (ASR) methods. The exact methods for data extraction have been described elsewhere [37]. The set of extracted acoustic features included more than 200 variables for each speech recording. A priori, we selected 11 acoustic features based on previously reported relevance for AD [6, 13–15]. A list of the selected acoustic features is presented in Table 1.

**Table 1:** Selected acoustic speech features

Feature	Description
Long pauses	The number of unfilled pauses (silences) longer than 2 s divided by the audio length in seconds.
Medium pauses	The number of pauses of 1–2 seconds, divided by the audio length in seconds.
Pause duration	The duration of segments without a speech signal divided by total number of segments without any speech signal in seconds. Includes all segments without any speech signal (including < 150 milliseconds).
Pause-to-word ratio	The number of segments without any speech signal longer than 150 milliseconds divided by number of segments with a speech signal.
Phonation rate	The number of segments with a speech signal (in 50 milliseconds windows) over the total number of speech segments, irrespective of audio duration.
Audio duration	The total length of the audio sample in seconds.
Fundamental frequency	The mean of the sequence of fundamental frequency values extracted from the audio file in Hertz, using the Parselmouth library (equivalent to Praat method for computing fundamental frequency). The cutoff range is 70–620 Hz.
Intensity	The mean of the intensity curve (i.e., loudness), relative to 2*10^− 5^ Pascal (normative auditory threshold for a 1000-Hertz sine wave) in decibel.
Intensity variance	The variance of the intensity curve (i.e., loudness), relative to 2*10^− 5^ Pascal (normative auditory threshold for a 1000-Hertz sine wave) in decibel.
Local shimmer	The average absolute difference between the amplitudes of consecutive periods, divided by the average amplitude, in percentages.
Local jitter	The average absolute difference between consecutive periods, divided by the average period, in percentages.

**Fig. 1:**
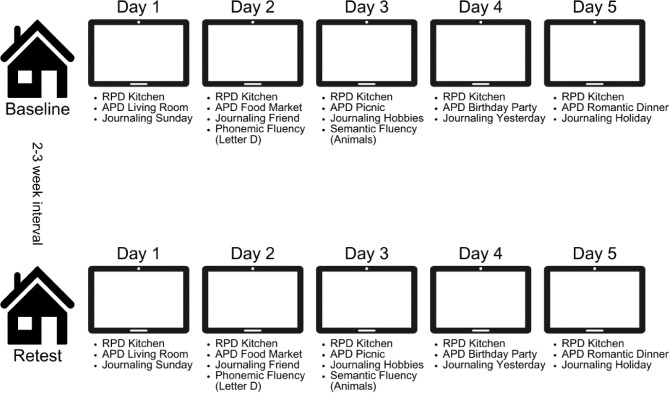
Procedure of Winterlight Assessment (WLA) app implemented in a multi-day testing design. *Note*: RPD = repetitive picture description; APD = alternating picture description

The speech assessment was incorporated into a multi-day testing design, where speech tasks were scheduled in a predetermined order across five days, as visualized in Fig. 1. The first assessment day was scheduled in accordance with the participant’s preference. Tasks could be completed any time between 06.00 AM and 00.00 PM. Participants were instructed to place the tablet nearby, and to complete all tasks of each assessment day at once, in a quiet environment without distractions. Participants received reminders from the research team (RB, MG) via email or by phone if two consecutive days were not completed. Daily administration time was approximately 5–10 minutes. The study protocol was repeated after 2–3 weeks to assess test-retest reliability.

After study enrollment, participants were provided login credentials for the WLA app by one of the researchers (RB, MG), either at the memory clinic of the Alzheimer Center Amsterdam, the participant’s home, or online via video-conferencing. Participants installed the WLA app on their own tablet (iOS), or they were given a study-provided tablet (iOS) with the WLA app already installed. Additionally, they were familiarized with the app interface by one of the researchers (RB, MG), where participants were shown a picture description task and journaling task in the WLA app, and where it was explained how to login in the WLA app, and how to exit the WLA app, which took approximately two to five minutes. Thereafter, participants self-administered the speech assessment unsupervised in their home environment (i.e., remotely). Test instructions in Dutch were both visually presented on screen, and auditorily provided by a computer-generated voice within the WLA app. The internal microphone of the device recorded the participant’s speech during task completion.

#### Feasibility and usability

Feasibility of the multi-day testing protocol was evaluated for the baseline speech assessment by evaluating drop-outs, adherence rates, rates of fully completed assessment days and the rate of errored speech samples. Drop-outs were defined as the number of participants who withdrew from the study before the close-out visit. Adherence rates were determined for the baseline multi-day speech assessment, by calculating the number of fully completed assessment days (i.e., all scheduled tasks completed) divided by the total number of five scheduled assessment days. For instance, completion of four out of the five consecutive days resulted in an adherence rate of 80%. In addition, to determine how many completed days are feasible to obtain in multi-day testing protocols, we calculated the number of participants who fully completed one up to five assessment days across the multi-day speech assessment. Moreover, we explored the rate of errored samples (e.g., poor quality or technical issues with speech samples), by dividing the number of errored samples by the total amount of collected speech samples.

To evaluate the usability of the speech assessment, we used a Dutch translation of the validated System Usability Scale (SUS) [38–40]. The SUS questionnaire consists of ten items containing statements such as “I thought the app was easy to use”. These statements were evaluated by respondents on a 5-point Likert scale ranging from strongly disagree to strongly agree. The SUS was completed by the participants after completion of the speech assessment. Based on the responses to individual statements, a total SUS score was calculated using a standard scoring procedure [39]. SUS-scores range from 0 to 100, where scores ≥ 71.4 are perceived to reflect good, and scores ≥ 85.5 excellent usability [41].

### Statistical analysis

Statistical analyses were performed using R (version 4.2.1). Participant characteristics were compared between Aβ-positive and Aβ-negative groups, using chi-square tests for categorical variables and two samples t-tests for continuous variables. If normality could not be assumed, the non-parametric Wilcoxon test was used, and if equality of variance could not be assumed, the Welch test was used.

To assess test-retest reliability in the total group, intraclass correlation coefficients (ICC) were computed between the baseline and retest speech assessments of each acoustic feature for each subtask separately. ICCs were computed between the provided speech samples of the baseline assessment and the provided speech samples of the retest assessment. To determine whether averaging over multi-day speech samples enhanced reliability, we additionally calculated ICCs for cumulative speech samples (i.e., between the mean score of two, three, four or five speech samples of the baseline and retest assessment). ICCs < 0.5 were considered as poor reliability, ICCs 0.5–0.75 as moderate reliability, ICCs 0.75–0.90 as good reliability, and ICCs > 0.90 as excellent reliability [42].

Furthermore, we investigated differences in each of the eleven acoustic speech characteristics between Aβ-positive and Aβ-negative individuals, thereby assessing differences in the mean and intra-individual variability. First, differences in mean scores between Aβ-groups were investigated using linear regression models (LM). LMs included Aβ-biomarker status as a predictor of interest, and acoustic speech parameters as outcome, adjusted for age, sex and years of education. Analyses were performed for each subtask and acoustic feature separately. Secondly, we examined group differences in intra-individual variability within speech acoustics using LMs with the same model structure as described above. Intra-individual variability was defined as the mean absolute deviation from the individual mean across the completed sessions of the baseline assessment and was calculated for each acoustic feature and speech task separately. We applied the false discovery rate (FDR) method to correct for multiple testing. For the remainder, p values < 0.05 were considered significant.

## Results

### Participant characteristics

An overview of demographics of the *N* = 50 participants is displayed in Table 2. On average, participants were 68.4 ± standard deviation (SD) 6.2 years of age (range 53–79), 58.0% (*n* = 29) was female, and the mean years of education was 15.3 ± 3.8 (range 9–25). The mean Mini-Mental State Examination (MMSE) score was 29.2 ± 1.0 (range 26–30). 23 (46%) participants were Aβ-positive. Aβ-groups did not differ in age, sex and years of education, MMSE scores were higher for the Aβ-positive (M = 29.5 ± 0.7, range 28–30) than for the Aβ-negative group (M = 28.8 ± 1.07, range 26–30, *p* = 0.012).

**Table 2:** Participant characteristics

	Total group (*N* = 50)	Amyloid-beta positive (*n* = 23)	Amyloid-beta negative (*n* = 27)	*p*-value
Age, years, mean ± SD	68.4 ± 6.2	69.6 ± 6.3	67.3 ± 6.0	0.193^a^
Female, n (%)	29 (58.0)	13 (56.5)	16 (59.3)	0.845^d^
Education, years, mean ± SD	15.3 ± 3.8	15.2 ± 4.6	15.3 ± 3.0	0.944^b^
Cohort				0.233^d^
AMYPAD PNHS, n (%)	34 (68.0)	13 (56.5)	21 (77.8)	
SCIENCe, n (%)	12 (24.0)	8 (34.8)	4 (14.8)	
ADC, n (%)	4 (8.0)	2 (8.7)	2 (7.4)	
Amyloid-beta biomarkers				0.508^d^
Cerebrospinal Fluid, n (%)	5 (10.0)	3 (13.0)	2 (7.4)	
Positron Emission Tomography, n (%)	45 (90.0)	20 (87.0)	25 (92.6)	
Mini-Mental State Examination, mean ± SD	29.2 ± 1.0	28.8 ± 1.1	29.5 ± 0.7	0.012^c^

*Note* Data are depicted as mean ± standard deviation (SD) unless otherwise indicated; Differences between amyloid-beta positive individuals and amyloid-beta negative individuals are tested . ^a^Student t-test, ^b^Welch t-test, ^c^ Wilcoxon test, ^d^Chi-Square test

### Feasibility and usability

Fifty participants provided a total of 784 (92.2%) out of 850 scheduled speech samples for the baseline multi-day assessment, and none of the participants dropped out. Across the baseline assessment that consisted of five days, the mean number of completed days was 4.6 (SD = 0.9, range 1–5), corresponding to a mean adherence rate of 91.6% (SD = 17.2, range 20.-100%). All participants (100% ) completed at least one assessment day. The majority also completed two (*n* = 49, 98.0%), three (*n* = 48, 96.0%) and four (*n* = 45, 90.0%) days, and 37 participants (74.0%) completed all five scheduled assessment days. Of the 784 collected baseline speech samples, 21 (2.7%) samples could not be further processed because of quality issues or technical issues with the speech sample (e.g., inaudible participant, no participant, incomplete file, invalid audio, corrupted file or administration issue). Supplementary Table 1 shows numbers of speech samples included for the baseline and retest multi-day speech assessments. Regarding the practical administration, the majority of the participants (*n* = 29, 58.0%) used a study-provided tablet.

**Fig. 2:**
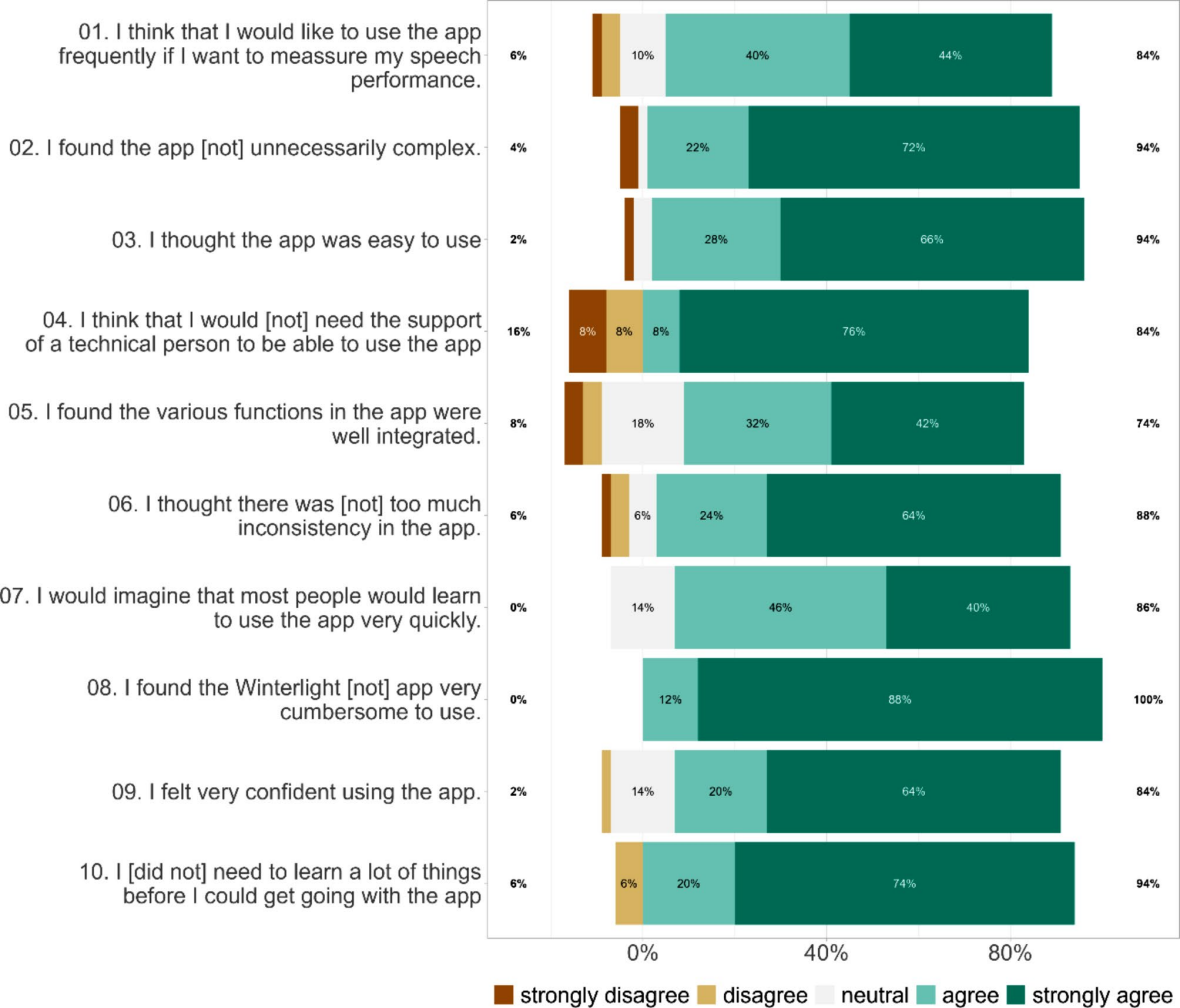
Responses on individual items of the System Usability Scale (SUS) in the total group. *Note*: Negatively phrased SUS-items (even-numbered) and their responses are reversed for visualization reasons, such that for all SUS-items agree-responses (green) indicate positively perceived usability

The usability of the speech assessment was evaluated by participants with a mean SUS-score of 86.0 ± 9.9 (range 55–100, median = 87.5), which was above the cut-off of 85.5, reflecting excellent usability [41]. Responses on the SUS-items are visualized in Fig. 2, where it can be observed that responses to individual SUS-items were largely uniform among participants.

### Test-retest reliability

ICCs were computed between the baseline and retest assessment for cumulative numbers of speech samples. Overall, ICCs ranged from − 0.06 to 0.97, depending on speech feature, number of averaged speech samples and subtask. In Supplementary Table 2 ICCs are shown.

**Fig. 3:**
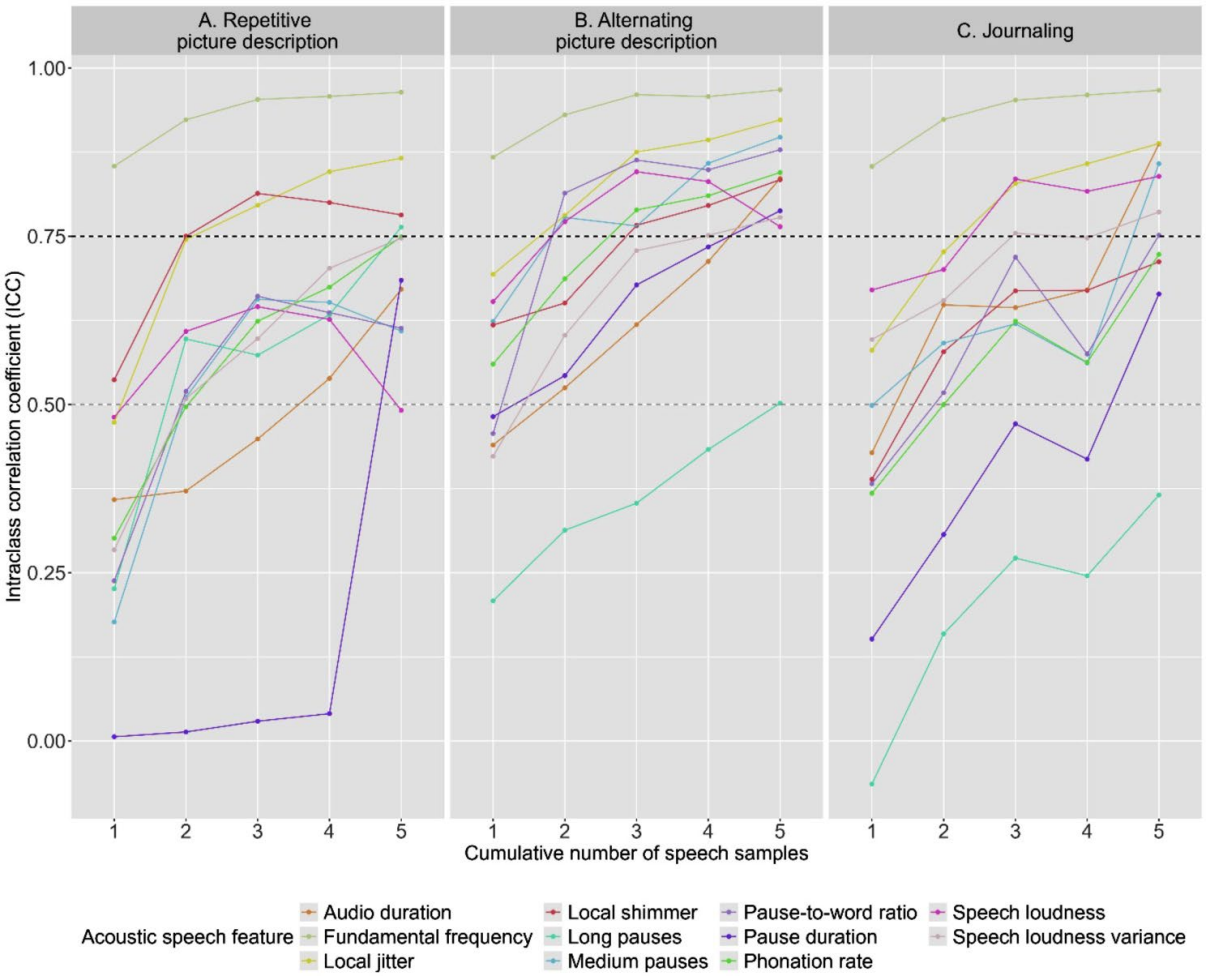
Intraclass correlation coefficients (ICCs) for test-retest reliabilities (2–3 week interval) for averaged acoustic speech features across cumulative numbers of sessions for (**A**) repetitive picture description, (**B**) alternating picture description and (**C**) journaling. *Note*: Grey dashed line indicates ICC ≥ 0.50 (moderate reliability), black dashed line indicates ICC ≥ 0.75 (good reliability). Note that ICCs were computed for cumulative numbers of averaged speech samples between the baseline and retest assessment

Regarding the multi-day testing protocol, the trend across all speech tasks was observed that ICCs increased with the number of averaged speech samples, as visualized in Fig. 3. In averaged measures across two speech samples, ICCs ≥ 0.50 (moderate reliability) were reached for all speech features, except for pause duration in repetitive picture description and journaling, and total audio duration in repetitive picture description. Focusing on the number of averaged samples needed to reach ICCs ≥ 0.75 (good reliability), overall less alternating picture description samples were needed than repetitive picture description and journaling samples. Specifically, in two samples of alternating picture description ICCs ≥ 0.75 were reached for five (45.5%) features, while in two samples of repetitive picture description and journaling this level was reached for respectively three (27.3%) and one (9.1%) of the features.

Zooming in on individual features, fundamental frequency was the only feature that had ICCs ≥ 0.75 in one speech sample. Jitter was measured with ICCs ≥ 0.75 if two picture description samples (repetitive or alternating), or three journaling samples were averaged. To reach good reliability for shimmer, two averaged repetitive or three averaged alternating picture description samples were needed. Medium pauses and pause-to-word ratio required two averaged alternating picture description or five averaged journaling samples. Intensity was measured with ICCs ≥ 0.75 if two alternating picture description samples or three journaling samples were averaged. This reliability level was reached for intensity variance after three, four or five averaged samples of journaling, alternating or repetitive picture description respectively. Phonation rate was measured with ICCs ≥ 0.75 in five repetitive or three alternating picture description samples. Audio duration required five averaged samples of alternating picture description or journaling. Long pauses and pause duration were measured with ICCs ≥ 0.75 in five averaged samples of averaged repetitive or alternating picture description respectively. Thus, overall ICCs increased with number of averaged sessions, such that all features could be measured with good reliability, although it differed for each feature what task and how many averaged samples were required. Based on the optimal trade-off between feasibility (i.e., four fully completed assessment days available for 90% of participants) and reliability (i.e., reliability increased with number of averaged speech samples), we decided to perform further analyses for speech features in averaged speech samples across four sessions.

### Differences in acoustic speech features between Aβ-positive and Aβ-negative groups

**Fig. 4:**
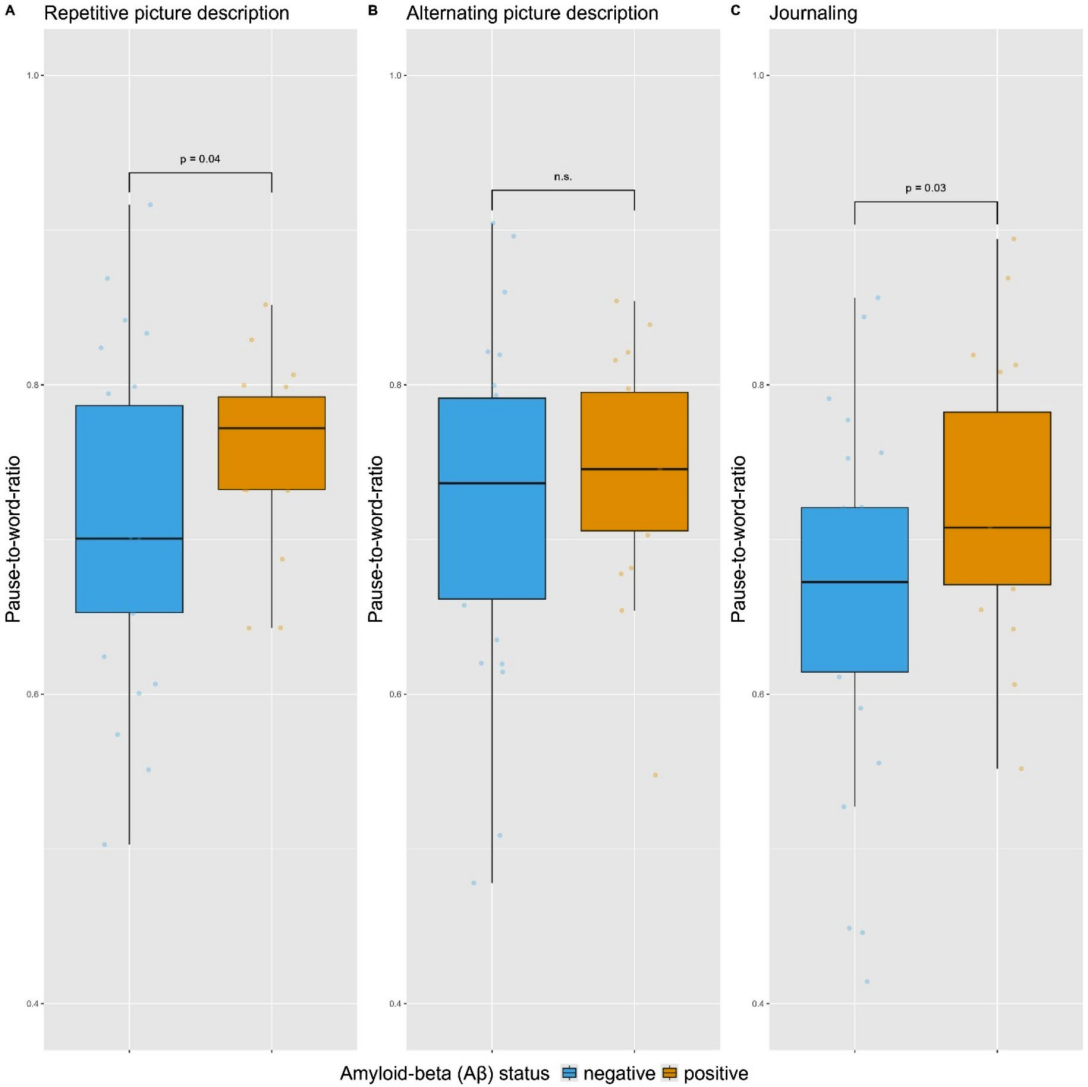
Pause-to-word ratio in Aβ-negative and Aβ-positive individuals for four sessions of (**A**) repetitive picture description, (**B**) alternating picture description and (**C**) journaling (averaged across four speech samples). *Note*: Data points represent unadjusted scores of the pause-to-word ratio for each individual participant. A higher pause-to-word ratio indicates a relatively higher number of pauses in speech production. The box represents the Interquartile Range (IQR) from the first (Q1) to third quartile (Q3), whiskers represent the minimum (Q1–1.5*IQR) and maximum (Q3 + 1.5*IQR) score, and the center line represents the median. Displayed p-values are values obtained from linear regression models assessing the differences between Aβ-positive and Aβ-negative individuals in acoustic speech features in four averaged speech samples adjusted for age, sex and education, and are not corrected for multiple testing; n.s. indicates not significant

We compared Aβ-groups on each acoustic speech feature in each subtask separately. Uncorrected analyses (i.e., not corrected for multiple testing) showed differences between Aβ-positive and Aβ-negative groups for pause-to-word ratio in the repetitive-PD subtask (*B* = 0.05, *95%CI* = 0.00–0.10, *p* = 0.040) and the journaling subtask (*B* = 0.07, *95%CI* = 0.01–0.13, *p =* 0.032), indicating that the speech production of Aβ-positive cognitively unimpaired individuals contained relatively more pauses than that of Aβ-negative individuals, which is visualized in Fig. 4. For none of the other acoustic features significant group differences were found in any of the speech subtasks (*p’s* > 0.05). Results of LMs are shown in Table 3, and mean scores are displayed in Supplementary Table 3. After correction for multiple testing, none of the differences between Aβ-groups in acoustic features reached significance (*p*’s > 0.05). Although acoustic speech features did not differ significantly between the Aβ-groups after correction for multiple comparisons, across speech tasks the overall pattern was observed that differences in acoustic features were consistently in the same direction, as visualized in Supplementary Fig. 1. Specifically, in all subtasks the Aβ-positive group had a higher score than the Aβ-negative group on intensity variance, pause-to-word ratio, medium pauses, local jitter, fundamental frequency and audio duration. The Aβ-positive group scored consistently lower than the Aβ-negative group on phonation rate, long pauses and local shimmer, and in two of the three subtasks on intensity and pause duration.

**Table 3:** Results of linear regression models (LMs) assessing differences between Aβ-positive and Aβ-negative individuals in acoustic speech features in four averaged speech samples, adjusted for age, sex and education

Features	Repetitive picture description		Alternating picture description		Journaling	
	Unadjusted estimate [95% CI]	Adjusted estimate [95% CI]	Unadjusted estimate [95% CI]	Adjusted estimate [95% CI]	Unadjusted estimate [95% CI]	Adjusted estimate [95% CI]
**Long pauses**	0.00 [-0.01–0.01]	-0.00 [-0.01–0.01]	-0.00 [-0.01–0.01]	-0.00 [-0.01–0.01]	-0.00 [-0.01–0.01]	-0.00 [-0.01–0.00]
**Medium pauses**	0.02 [-0.02–0.05]	0.01 [-0.03–0.05]	0.01 [-0.03–0.05]	0.01 [-0.03–0.05]	0.03 [-0.02–0.09]	0.02 [-0.04–0.08]
**Pause word ratio**	0.05 [-0.00–0.10]	**0.05** **[0.00–0.10]**	0.02 [-0.03–0.08]	0.03 [-0.03–0.08]	0.06 [-0.00–0.13]	**0.07** **[0.01–0.13]**
**Pause duration**	-0.56 [-1.82–0.70]	-0.15 [-1.47–1.18]	0.02 [-0.11–0.15]	0.01 [-0.12–0.15]	-0.00 [-0.17–0.17]	-0.03 [-0.20–0.13]
**Phonation rate**	-0.02 [-0.09–0.05]	-0.02 [-0.10–0.05]	-0.01 [-0.07–0.04]	-0.01 [-0.06–0.04]	-0.02 [-0.08–0.04]	-0.00 [-0.07–0.06]
**Total audio duration**	15.79 [-15.24–46.82]	16.56 [-16.79–49.91]	12.29 [-17.44–42.02]	10.62 [-21.38–42.61]	1.18 [-18.35–20.71]	2.74 [-18.10–23.59]
**Fundamental frequency**	5.13 [-14.48–24.75]	7.62 [-4.91–20.16]	4.87 [-15.31–25.05]	6.10 [-7.06–19.25]	5.47 [-13.93–24.87]	6.75 [-6.06–19.55]
**Intensity**	0.83 [-1.68–3.34]	0.49 [-2.21–3.19]	-0.41 [-3.08–2.26]	-0.80 [-3.65–2.05]	0.02 [-2.58–2.62]	-0.28 [-3.08–2.52]
**Intensity variance**	18.59 [-11.71–48.89]	13.02 [-19.04–45.08]	9.63 [-18.10–37.36]	4.40 [-24.76–33.56]	10.56 [-16.65–37.78]	3.30 [-24.78–31.37]
**Local shimmer**	-0.01 [-0.10–0.08]	-0.02 [-0.10–0.06]	-0.01 [-0.09–0.08]	-0.01 [-0.09–0.07]	-0.00 [-0.08–0.08]	-0.00 [-0.09–0.08]
**Local jitter**	0.00 [-0.00–0.00]	0.00 [-0.00–0.00]	0.00 [-0.00–0.00]	0.00 [-0.00–0.00]	0.00 [-0.00–0.00]	0.00 [-0.00–0.00]

*Note* 95% CI indicates 95% confidence interval. Analyses are not corrected for multiple comparisons. Significant effects are in bold

**Fig. 5:**
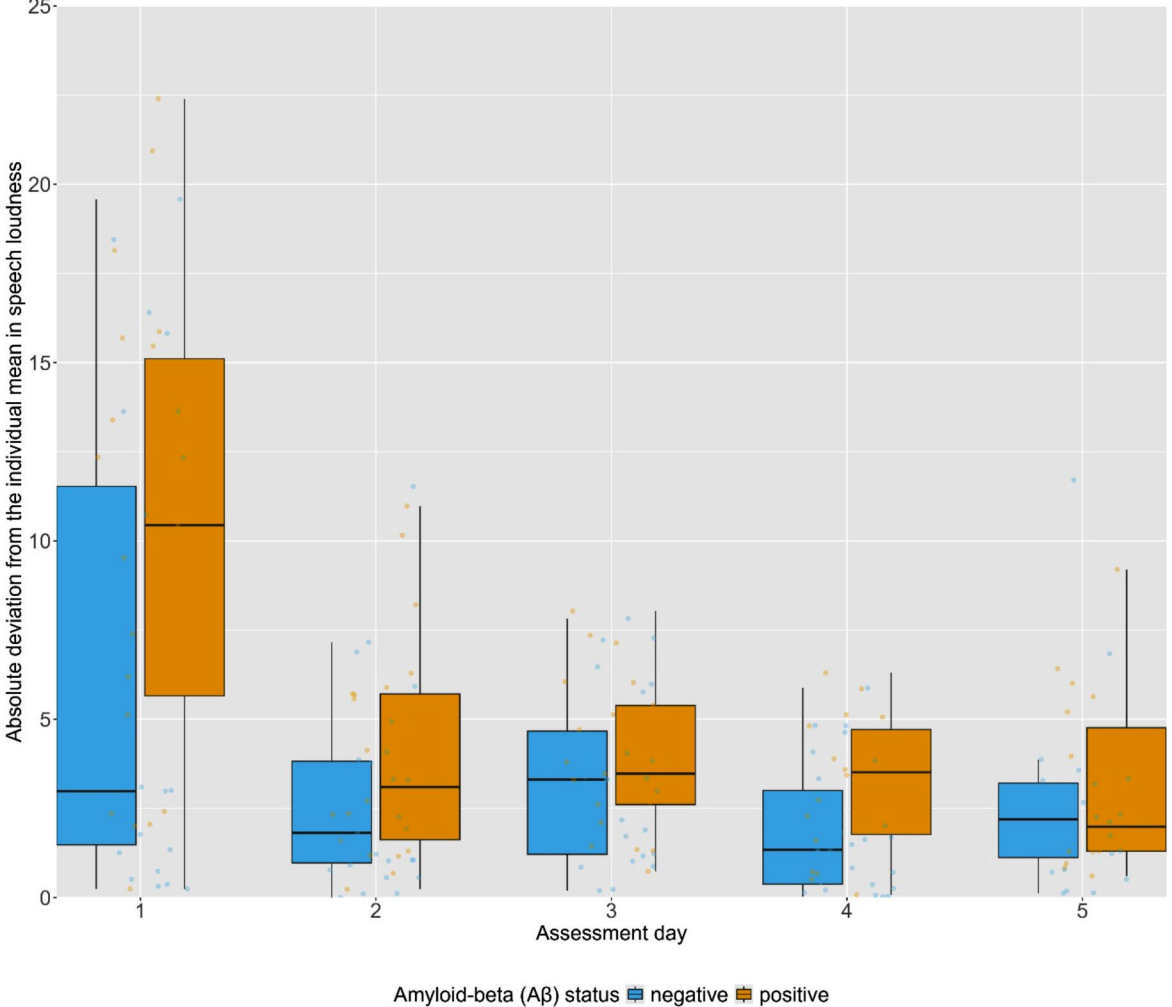
Absolute deviation from the individual mean in mean intensity for each repetitive-PD session in Aβ-negative and Aβ-positive groups. *Note*: Data points represent unadjusted scores of the absolute deviation from the individual mean for each individual participant. The box represents the Interquartile Range (IQR) from the first (Q1) to third quartile (Q3), whiskers represent the minimum (Q1–1.5*IQR) and maximum (Q3 + 1.5*IQR) score, and the center line represents the median

Regarding intra-individual variability (IIV) in the acoustic speech features, across the repetitive-PD sessions the mean intra-individual variability in intensity was higher in the Aβ-positive group (M_IIV_ = 5.11 ± 2.41) than in the Aβ-negative group (M_IIV_ = 3.35 ± 2.58, *B* = 1.84, 95% CI = 0.33–3.35, *P* = 0.018). The intra-individual variability in intensity across the repetitive-PD sessions is visualized in Fig. 5. For none of the other acoustic features significant group differences in intra-individual variability were found in any of the subtasks (*p’s* > 0.05, see Supplementary Table 4). After adjusting for multiple comparisons, none of the Aβ-group differences in intra-individual variability reached significance (*p’s* > 0.05).

## Discussion

This study showed that remote assessment of connected speech production is a feasible and reliable method to assess acoustic speech features in preclinical AD. We found that a higher pause-to-word ratio distinguished cognitively unimpaired individuals with Aβ-positive biomarkers from individuals with negative Aβ-biomarkers, although significance was lost after correction for multiple testing. These results underline the potential of remotely measured speech acoustics over multiple assessments as a promising indicator of subtle cognitive deficits in early AD stages.

The speech assessment was shown to be feasible, both from the participant perspective (i.e., high adherence) and the technical processing perspective (i.e., few quality or technical issues with speech samples). Adherence rates for remote multi-day cognitive assessments have previously been reported to be high in groups with varying cognitive status (i.e., cognitively unimpaired, MCI, mild dementia), where mean or median adherence ranged from 80-93%^43–45^. Our findings of overall 91.6% adherence is in line with these previous reports, and indicates that older adults, also those who are worried about their cognition and therefore visited the memory clinic, are motivated to engage in studies using remote assessments. Although assessments were unsupervised, technical assistance was available when needed, and participants received reminders if two consecutive days were not completed. This level of (technical) support might have enhanced adherence, and underlines previously identified preferences from end users that support staff is a desirable aspect of remote cognitive assessment [46]. Accordingly, when designing remote testing protocols, access to remote assistance should be provided. Moreover, usability of the speech assessment was excellent, consistent with previous reports that indicated good usability for other self-administered tablet-based cognitive assessments [46–48]. Familiarity with application interfaces might have partially motivated our high usability evaluations, as we only included participants who self-reported to have experience with such devices, although previous research has shown that usability did not depend on device familiarity [47]. Hence, these high usability ratings support the use of remote tablet-based cognitive assessments for older adults.

Regarding the reliability of acoustic speech features, no consensus has been reached within the current literature, although previous studies have reported low to high reliability for pausing features [19, 49, 50], moderate reliability for jitter and shimmer [51, 52], and high reliability for fundamental frequency [50, 51]. Our findings contribute to this body of literature, demonstrating that most acoustic speech features showed relatively low reliability if measured in only a single speech sample, but reliability improved significantly when averaged across multiple speech samples. This trend was irrespective of outcome feature or speech task, such that all acoustic features could be measured with good reliability. As such, our findings support the view that averaged assessments offer a more reliable index of cognitive performance than one-occasion testing [43, 44, 53]. This need for repeated assessment to acquire high reliability of acoustic speech features may not be surprising, given that spontaneous speech is an inherently unstructured outcome measure, characterized by variations, that is thus difficult to capture reliably with a single assessment. Regarding specific speech tasks, alternating picture description required overall fewer averaged samples than repetitive picture description and journaling, suggesting that the former task is the most reliable measure of speech acoustics. Although more consistency might have been expected for repeated descriptions of the same picture, it might be speculated that participants were less engaged to describe the same picture multiple times, resulting in relatively lower reliability levels for repetitive than alternating picture description. The relatively lower reliability in the journaling task may be driven by the less structured nature of this task, such that more averaged samples were required to obtain good reliability. It should be noted, however, that with increased number of completed assessment days, adherence decreased, where up to four assessment days were feasible to complete most participants. This trade-off between feasibility and reliability should be considered in the design of repeated testing protocols.

We observed a trend that the speech of Aβ-positive individuals was characterized by more pauses (i.e., higher pause-to-word ratio) than that of Aβ-negative individuals in repetitive picture description and journaling, which is in line with current literature that pausing features are among the most important acoustic features associated with AD pathology [15]. An increased use of pauses has previously been suggested to reflect different underlying processes, such as difficulties with lexical retrieval, episodic memory or planning [7, 54–57], that may thus be evident as early as in the preclinical AD stage. As such, speech may serve as a window to underlying cognitive processes. The underlying cognitive processes that are required may differ between speech tasks, as may the cognitive load associated with each speech task. Accordingly, such differences in cognitive demands may explain why the most pronounced Aβ-related acoustic differences were observed in journaling and repetitive picture description, rather than in alternating picture description. Narrative tasks, such as journaling and picture description, require executive functioning processes such as planning and organization, in order to produce a well-structured narrative [58]. Journaling may be argued to place higher demands on executive functioning processes than picture description, as no cues such as pictures are provided in this task. The two speech tasks may also differ regarding lexical retrieval processes, where the provided image in the picture description task might activate lexical concepts, thereby possibly facilitating lexical retrieval [59, 60]. Moreover, journaling questions prompted participants to retell events from the past, thereby placing demands on episodic memory. The repetitive and alternating picture description tasks may differ in the demands placed on memory recall, that might be required by the former task (“What did I say about the picture yesterday?”), possibly resulting in more pauses, whereas the latter task does not do so specifically. Accordingly, tasks placing higher loads on the cognitive system are potentially more sensitive to detect AD-related acoustic deviations in speech, as previously suggested [15].

Moreover, as intra-individual variability has been suggested as a promising cognitive marker of AD itself [45, 61], although not universally reported in the literature [43], we assessed variability in speech acoustics over multiple days. In the Aβ-positive group, intensity fluctuated to a higher extent over days for the repetitive picture descriptions. To the best of our knowledge, such an observation of fluctuations in intensity over days has not been described in previous literature. Still, this finding may support the previous suggestion that higher intra-individual variability might reflect subtle cognitive decline. It should be noted though that participant-tablet interactions may interfere with recording of intensity, such as the distance between the speaker and tablet fluctuating across days [62]. This may especially have occurred since we did not provide instructions regarding the speaker-to-microphone distance, and as such it is recommended to include such instructions in future remote speech assessment protocols.

Our study has several strengths and limitations. The primary strength was that our study sample of cognitively unimpaired adults was well-phenotyped with clinical data and Aβ-biomarkers. Additionally, by performing the study in a home-based environment, the ecological validity of our speech task was high. Another strength, in this context, was that we used rather unstructured speech tasks to elicit speech. As such, the provided speech samples were representative of everyday language use, thereby providing insight in the characterization of the acoustic speech profile of semi-spontaneous speech in the preclinical AD stage. A limitation regarding the unsupervised home-based setting, however, was that we could not control for distractions, background noise and microphone distance while testing, which may have affected the quality of the speech recordings. We acknowledge that some acoustic features may be susceptible to noise in the audio signal caused by the uncontrolled, remote setting that does thus not provide the ideal acoustic environment. Specifically, measures of jitter and shimmer have previously been shown to have limited reliability [52, 63]. The aim of this study, however, was to evaluate the feasibility and reliability of measuring speech acoustics given this uncontrolled, remote environment by using multi-day assessments The limitations inherent to unsupervised remote testing in an uncontrolled setting should be acknowledged as challenges of remote assessment in general, and should be minimized in future research by providing clear testing instructions regarding the testing environment and device placing distance. We argue, however, that given the multi-day paradigm we used, such influences of the testing environment on test performance are probably reduced to some extent. Another limitation is that the study sample was relatively small, limiting the generalizability of our results. In addition, we did not consider potential effects depression, autism, or dialects, that could have influenced acoustic speech characteristics, and these associations should thus be assessed in future studies.

In this study we demonstrated the feasibility and test-retest reliability of remote assessment of acoustic speech features in the at-home environment, which are essential validation steps towards the application of remote acoustic speech biomarkers in clinical practice. Since acoustic analysis of the raw audio signal is largely language-independent, and does not require manual transcriptions, acoustic speech biomarkers offer a non-invasive, time-efficient and therefore scalable method, that have high potential for remote monitoring in for example decentralized trials. As we demonstrated associations between remotely measured speech acoustics and Aβ-pathology, this may indicate that such speech features could indeed be sensitive to Aβ-related change over time. Therefore, future research should assess longitudinal relationships between Aβ-pathology and acoustic speech features. Additionally, further research should assess the relationship between Aβ-pathology and remotely obtained linguistic content characteristics of speech (i.e., at the lexical, semantic and syntactic level) in cognitively unimpaired individuals, to provide further insight in the speech profile of individuals with preclinical AD.

### Electronic supplementary material

Below is the link to the electronic supplementary material.

Supplementary Material 1

## Data Availability

The data that support the findings of this study are available from the corresponding author upon reasonable request.
