# Supplementary material for: Digital remote assessment of speech acoustics in cognitively unimpaired adults: feasibility, reliability and associations with amyloid pathology
Source: Alzheimers Res Ther. 2024 Aug 1;16:176. doi: 10.1186/s13195-024-01543-3 (PMC11293000; doi:10.1186/s13195-024-01543-3)
Supplement: Supplementary file 1 — Supplementary Material 1 [file 13195_2024_1543_MOESM1_ESM.docx]

# **SUPPLEMENTAL MATERIAL**

### **Supplementary Table 1.** Total number of speech samples included in analyses for the baseline and retest multi-day speech assessment for each subtask and each assessment day.

|  | Baseline multi-day speech assessment (N = 763) | Retest multi-day speech assessment (N = 730) |
| --- | --- | --- |
| Repetitive picture description | 232 | 220 |
| Day 1 | 50 | 44 |
| Day 2 | 49 | 42 |
| Day 3 | 45 | 46 |
| Day 4 | 44 | 45 |
| Day 5 | 44 | 43 |
| Alternating picture description | 229 | 219 |
| Day 1 | 49 | 43 |
| Day 2 | 49 | 42 |
| Day 3 | 44 | 46 |
| Day 4 | 43 | 44 |
| Day 5 | 44 | 44 |
| Journaling | 217 | 209 |
| Day 1 | 47 | 41 |
| Day 2 | 46 | 41 |
| Day 3 | 41 | 45 |
| Day 4 | 43 | 40 |
| Day 5 | 40 | 42 |
| Phonemic fluency (Day 2) | 46 | 38 |
| Semantic fluency (Day 3) | 39 | 44 |
| Total errored samples | 21 | 20 |

### **Supplementary Table 2.** Intraclass correlation coefficients for test-retest reliabilities (2-3 week interval) and corresponding level of significance for acoustic speech features in cumulative numbers of assessments.

| **Number of averaged assessments** | **N_observations_** | **Long pauses** | **Medium pauses** | **Pause-to-word ratio** | **Pause duration** | **Phonation rate** | **Audio duration** | **Fundamental frequency** | **Intensity** | **Intensity variance** | **Local shimmer** | **Local jitter** |
| --- | --- | --- | --- | --- | --- | --- | --- | --- | --- | --- | --- | --- |
| Repetitive picture description |  |  |  |  |  |  |  |  |  |  |  |  |
| 1 assessment | 50 | 0.23 | 0.18 | 0.24* | 0.01 | 0.30* | 0.36** | **0.85****** | 0.48*** | 0.28* | 0.54**** | 0.47**** |
| 2 assessments | 49 | 0.60**** | 0.51**** | 0.52**** | 0.01 | 0.50**** | 0.37*** | **0.92****** | 0.61**** | 0.51*** | **0.75****** | **0.75****** |
| 3 assessments | 47 | 0.57**** | 0.66**** | 0.66**** | 0.03 | 0.62**** | 0.45*** | **0.95****** | 0.65**** | 0.60**** | **0.81****** | **0.80****** |
| 4 assessments | 42 | 0.63**** | 0.65**** | 0.64**** | 0.04 | 0.67**** | 0.54**** | **0.96****** | 0.63**** | 0.70**** | **0.80****** | **0.85****** |
| 5 assessments | 24 | **0.76****** | 0.61*** | 0.61**** | 0.68**** | **0.75****** | 0.67*** | **0.96****** | 0.49** | **0.75****** | **0.78****** | **0.87****** |
| Alternating picture description |  |  |  |  |  |  |  |  |  |  |  |  |
| 1 assessment | 49 | 0.21 | 0.62**** | 0.46**** | 0.48**** | 0.56**** | 0.44*** | **0.87****** | 0.65**** | 0.42*** | 0.62**** | 0.69**** |
| 2 assessments | 48 | 0.31* | **0.78****** | **0.81****** | 0.54**** | 0.69**** | 0.52**** | **0.93****** | **0.77****** | 0.60**** | 0.65**** | **0.78****** |
| 3 assessments | 46 | 0.35** | **0.77****** | **0.86****** | 0.68**** | **0.79****** | 0.62**** | **0.96****** | **0.85****** | 0.73**** | **0.77****** | **0.88****** |
| 4 assessments | 41 | 0.43*** | **0.86****** | **0.85****** | 0.73**** | **0.81****** | 0.71**** | **0.96****** | **0.83****** | **0.75****** | **0.80****** | **0.89****** |
| 5 assessments | 23 | 0.50*** | **0.90****** | **0.88****** | **0.79****** | **0.84****** | **0.84****** | **0.97****** | **0.76****** | **0.78****** | **0.83****** | **0.92****** |
| Journaling |  |  |  |  |  |  |  |  |  |  |  |  |
| 1 assessment | 49 | -0.06 | 0.50**** | 0.38*** | 0.15 | 0.37*** | 0.43*** | **0.85****** | 0.67**** | 0.60**** | 0.39*** | 0.58**** |
| 2 assessments | 48 | 0.16 | 0.59**** | 0.52**** | 0.31* | 0.50**** | 0.65*** | **0.92****** | 0.70**** | 0.65**** | 0.58**** | 0.73**** |
| 3 assessments | 46 | 0.27* | 0.62**** | 0.72**** | 0.47**** | 0.62**** | 0.64**** | **0.95****** | **0.84****** | **0.75****** | 0.67**** | **0.83****** |
| 4 assessments | 41 | 0.25* | 0.56**** | 0.58**** | 0.42*** | 0.56**** | 0.67**** | **0.96****** | **0.82****** | **0.75****** | 0.67**** | **0.86****** |
| 5 assessments | 23 | 0.37* | **0.86****** | **0.75****** | 0.66**** | **0.72****** | **0.89****** | **0.97****** | **0.84****** | **0.79****** | 0.71**** | **0.89****** |
| Phonemic fluency  1 assessment | 37 | 0.24 | 0.48*** | 0.28* | 0.35* | 0.44*** | 0.01 | **0.80****** | 0.56**** | 0.54**** | 0.46*** | 0.44*** |
| Semantic fluency  1 assessment | 37 | 0.30* | 0.37* | 0.20 | 0.31* | 0.40** | -0.21 | **0.90****** | 0.67**** | 0.46*** | 0.66**** | 0.65**** |

*Note*. * indicates P value <0.05, ** indicates P value <0.01, *** indicates P value <0.005, **** indicates P value <0.001. ICCs ≥ 0.75 are in bold.

### **Supplementary Table 3**. Mean scores and standard deviation (SD) on acoustic speech features in four averaged speech samples.

|  | **Repetitive picture description** |  | **Alternating picture description** |  | **Journaling** |  |
| --- | --- | --- | --- | --- | --- | --- |
|  | **Amyloid-positive**  **(n = 20)** | **Amyloid-negative**  **(n = 26)** | **Amyloid-positive**  **(n = 19)** | **Amyloid-negative**  **(n = 26)** | **Amyloid-positive**  **(n = 20)** | **Amyloid-negative**  **(n = 26)** |
| **Long pauses, hertz** | 0.02 (0.01) | 0.02 (0.02) | 0.02 (0.01) | 0.02 (0.02) | 0.01 (0.01) | 0.01 (0.02) |
| **Medium pauses, hertz** | 0.27 (0.06) | 0.25 (0.07) | 0.27 (0.06) | 0.26 (0.07) | 0.33 (0.09) | 0.29 (0.09) |
| **Pause word ratio, -** | 0.76 (0.05) | 0.71 (0.10) | 0.75 (0.07) | 0.72 (0.10) | 0.72 (0.09) | 0.66 (0.11) |
| **Pause duration, -** | 0.81 (0.25) | 1.36 (2.78) | 0.71 (0.20) | 0.69 (0.22) | 0.60 (0.19) | 0.60 (0.33) |
| **Phonation rate, -** | 0.74 (0.09) | 0.76 (0.13) | 0.80 (0.08) | 0.81 (0.10) | 0.79 (0.09) | 0.81 (0.11) |
| **Total audio duration, seconds** | 131.99 (55.57) | 116.19 (48.68) | 127.32 (51.02) | 115.03 (47.22) | 63.25 (33.86) | 62.07 (30.74) |
| **Fundamental frequency, hertz** | 156.42 (32.51) | 151.29 (32.88) | 155.95 (31.12) | 151.08 (34.54) | 151.06 (31.02) | 145.59 (32.47) |
| **Intensity, decibel** | 54.06 (4.26) | 53.23 (4.12) | 51.20 (4.34) | 51.60 (4.42) | 52.62 (4.09) | 52.60 (4.40) |
| **Intensity variance, decibel** | 184.89 (45.23) | 166.30 (54.24) | 169.20 (45.70) | 159.57 (45.46) | 145.78 (49.83) | 135.21 (40.62) |
| **Local shimmer, %** | 1.09 (0.14) | 1.10 (0.15) | 1.11 (0.14) | 1.11 (0.13) | 1.12 (0.14) | 1.12 (0.14) |
| **Local jitter, %** | 0.03 (0.01) | 0.03 (0.01) | 0.03 (0.00) | 0.03 (0.01) | 0.03 (0.00) | 0.03 (0.01) |


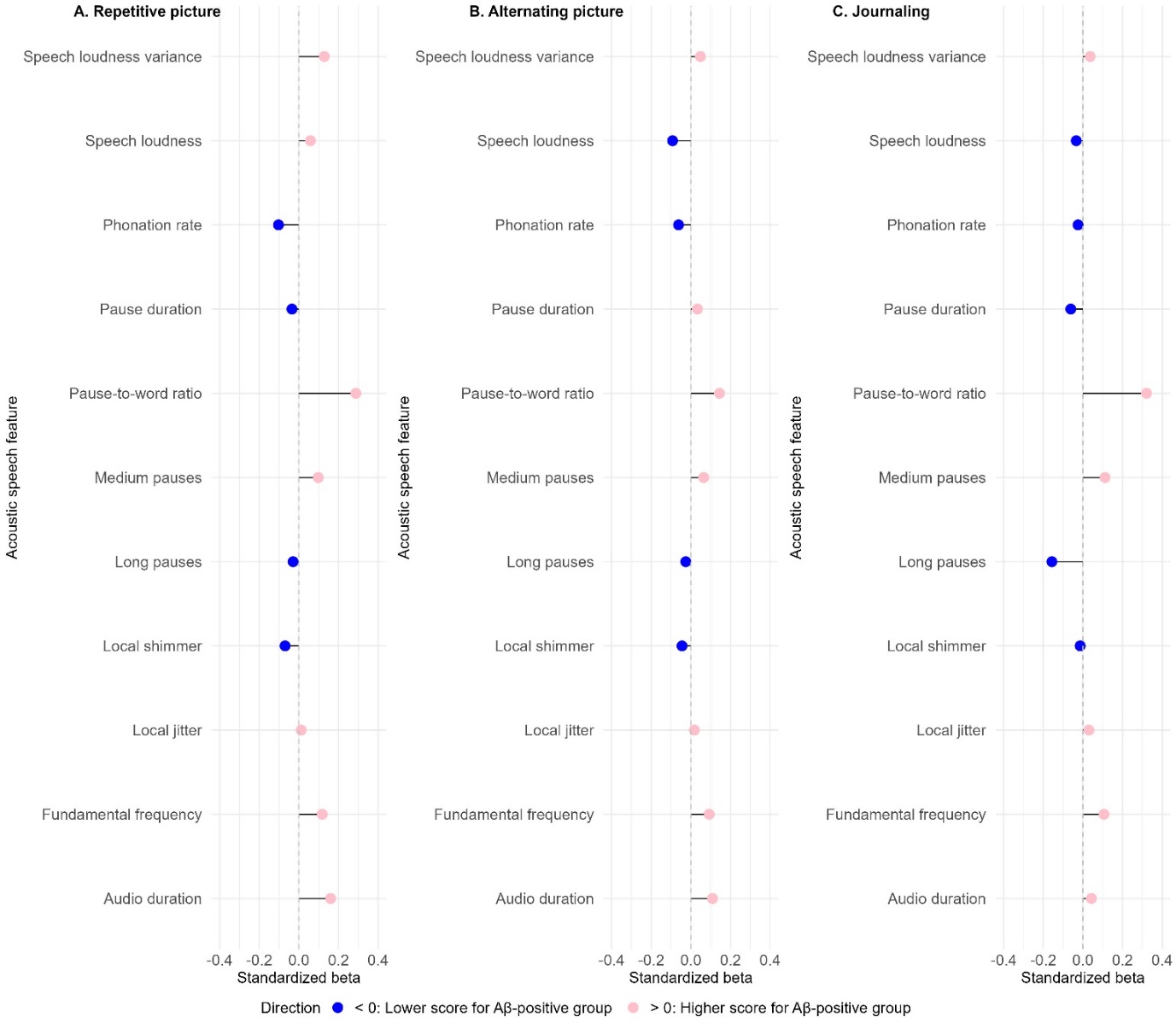


**Supplementary Figure 1.** Standardized betas from linear regression models (LMs) assessing differences between Aβ-positive and Aβ-negative individuals in acoustic speech features in (A) repetitive picture description, (B) alternating picture description and (C) journaling (averaged scores across four speech samples). LMs are corrected for age, sex and years of education.

*Note.* Standardized beta’s < 0 (blue) indicate lower scores for the Aβ-positive group compared to Aβ-negative group. Standardized beta’s > 0 (pink) indicate higher scores for the Aβ-positive group compared to Aβ-negative group. In all subtasks the Aβ-positive group had a higher score than the Aβ-negative group on intensity variance, pause-to-word ratio, medium pauses, local jitter, fundamental frequency and audio duration. The Aβ-positive group scored consistently lower than the Aβ-negative group on phonation rate, long pauses and local shimmer, and in two of the three subtasks on intensity and pause duration.

### **Supplementary Table 4.** Results of linear regression models (LMs) assessing differences between Aβ-positive and Aβ-negative individuals in intra-individual variability (IIV) in acoustic speech features across multi-day speech tasks, adjusted for age, sex and education.

1. **Repetitive picture description**

|  | **IIV Long pauses** |  | **IIV Medium pauses** |  | **IIV Pause-to-word ratio** |  | **IIV Pause duration** |  |
| --- | --- | --- | --- | --- | --- | --- | --- | --- |
| *Predictors* | *Estimates* | *95%CI* | *Estimates* | *95%CI* | *Estimates* | *95%CI* | *Estimates* | *95%CI* |
| (Intercept) | 0.02 | -0.01 – 0.04 | 0.03 | -0.07 – 0.13 | **0.15** | **0.04 – 0.27** | 8.12 | -2.00 – 18.24 |
| Amyloid status | -0.00 | -0.01 – 0.00 | -0.00 | -0.02 – 0.02 | -0.00 | -0.02 – 0.01 | -0.54 | -2.20 – 1.12 |
| Age | -0.00 | -0.00 – 0.00 | 0.00 | -0.00 – 0.00 | -0.00 | -0.00 – 0.00 | -0.13 | -0.29 – 0.02 |
| Sex | **-0.00** | **-0.01 – -0.00** | 0.00 | -0.02 – 0.02 | 0.00 | -0.02 – 0.02 | 0.26 | -1.52 – 2.04 |
| Education | -0.00 | -0.00 – 0.00 | 0.00 | -0.00 – 0.00 | -0.00 | -0.00 – 0.00 | 0.12 | -0.12 – 0.37 |
| Observations | 50 |  | 50 |  | 50 |  | 50 |  |
| R^2^ / R^2^ adjusted | 0.125 / 0.047 |  | 0.004 / -0.085 |  | 0.071 / -0.011 |  | 0.098 / 0.018 |  |

|  | **IIV Phonation rate** |  | **IIV Total audio duration** |  | **IIV Fundamental frequency** |  | **IIV Intensity** |  |
| --- | --- | --- | --- | --- | --- | --- | --- | --- |
| *Predictors* | *Estimates* | *95%CI* | *Estimates* | *95%CI* | *Estimates* | *95%CI* | *Estimates* | *95%CI* |
| (Intercept) | 0.18 | -0.07 – 0.43 | -37.80 | -98.89 – 23.29 | 6.16 | -4.92 – 17.24 | 5.53 | -3.65 – 14.72 |
| Amyloid status | -0.01 | -0.05 – 0.03 | 4.13 | -5.89 – 14.16 | 0.55 | -1.27 – 2.37 | **1.84** | **0.33 – 3.35** |
| Age | -0.00 | -0.01 – 0.00 | 0.72 | -0.21 – 1.66 | 0.02 | -0.15 – 0.19 | -0.04 | -0.18 – 0.10 |
| Sex | -0.01 | -0.05 – 0.04 | 3.46 | -7.29 – 14.21 | **2.26** | **0.31 – 4.21** | -0.16 | -1.78 – 1.45 |
| Education | 0.00 | -0.00 – 0.01 | 0.63 | -0.85 – 2.11 | -0.17 | -0.44 – 0.10 | 0.03 | -0.20 – 0.25 |
| Observations | 50 |  | 50 |  | 50 |  | 50 |  |
| R^2^ / R^2^ adjusted | 0.044 / -0.041 |  | 0.122 / 0.044 |  | 0.187 / 0.115 |  | 0.119 / 0.041 |  |

|  | **IIV Intensity variance** |  | **IIV Local shimmer** |  | **IIV Local jitter** |  |
| --- | --- | --- | --- | --- | --- | --- |
| *Predictors* | *Estimates* | *95%CI* | *Estimates* | *95%CI* | *Estimates* | *95%CI* |
| (Intercept) | 15.39 | -60.16 – 90.95 | 0.04 | -0.09 – 0.17 | 0.00 | -0.00 – 0.01 |
| Amyloid status | 7.79 | -4.61 – 20.19 | 0.02 | -0.01 – 0.04 | -0.00 | -0.00 – 0.00 |
| Age | -0.12 | -1.27 – 1.04 | -0.00 | -0.00 – 0.00 | 0.00 | -0.00 – 0.00 |
| Sex | 5.06 | -8.23 – 18.36 | 0.00 | -0.02 – 0.03 | 0.00 | -0.00 – 0.00 |
| Education | 1.03 | -0.80 – 2.86 | 0.00 | -0.00 – 0.00 | -0.00 | -0.00 – 0.00 |
| Observations | 50 |  | 50 |  |  |  |
| R^2^ / R^2^ adjusted | 0.065 / -0.018 |  | 0.064 / -0.019 |  |  |  |

1. **Alternating picture description**

|  | **IIV Long pauses** |  | **IIV Medium pauses** |  | **IIV Pause-to-word ratio** |  | **IIV Pause duration** |  |
| --- | --- | --- | --- | --- | --- | --- | --- | --- |
| *Predictors* | *Estimates* | *95%CI* | *Estimates* | *95%CI* | *Estimates* | *95%CI* | *Estimates* | *95%CI* |
| (Intercept) | 0.01 | -0.01 – 0.04 | 0.00 | -0.06 – 0.06 | 0.09 | -0.00 – 0.18 | 0.09 | -0.17 – 0.35 |
| Amyloid status | -0.00 | -0.01 – 0.00 | -0.01 | -0.02 – 0.00 | -0.00 | -0.02 – 0.01 | -0.02 | -0.07 – 0.02 |
| Age | -0.00 | -0.00 – 0.00 | 0.00 | -0.00 – 0.00 | -0.00 | -0.00 – 0.00 | 0.00 | -0.00 – 0.01 |
| Sex | -0.00 | -0.01 – 0.00 | -0.00 | -0.01 – 0.01 | 0.00 | -0.01 – 0.02 | -0.03 | -0.07 – 0.02 |
| Education | 0.00 | -0.00 – 0.00 | -0.00 | -0.00 – 0.00 | -0.00 | -0.00 – 0.00 | -0.00 | -0.01 – 0.00 |
| Observations | 50 |  | 50 |  | 50 |  | 50 |  |
| R^2^ / R^2^ adjusted | 0.103 / 0.023 |  | 0.060 / -0.024 |  | 0.048 / -0.037 |  | 0.076 / -0.006 |  |

|  | **IIV Phonation rate** |  | **IIV Total audio duration** |  | **IIV Fundamental frequency** |  | **IIV Intensity** |  |
| --- | --- | --- | --- | --- | --- | --- | --- | --- |
| *Predictors* | *Estimates* | *95%CI* | *Estimates* | *95%CI* | *Estimates* | *95%CI* | *Estimates* | *95%CI* |
| (Intercept) | 0.07 | -0.04 – 0.17 | 11.20 | -53.85 – 76.26 | -0.77 | -11.33 – 9.79 | **3.38** | **0.68 – 6.09** |
| Amyloid status | -0.00 | -0.02 – 0.01 | 1.68 | -8.99 – 12.36 | -0.27 | -2.00 – 1.47 | 0.18 | -0.26 – 0.62 |
| Age | -0.00 | -0.00 – 0.00 | 0.22 | -0.78 – 1.21 | 0.07 | -0.09 – 0.23 | -0.03 | -0.07 – 0.01 |
| Sex | -0.01 | -0.03 – 0.00 | 5.21 | -6.24 – 16.66 | **2.57** | **0.71 – 4.43** | 0.15 | -0.33 – 0.62 |
| Education | 0.00 | -0.00 – 0.00 | -0.26 | -1.83 – 1.32 | 0.05 | -0.21 – 0.30 | -0.00 | -0.07 – 0.06 |
| Observations | 50 |  | 50 |  | 50 |  | 50 |  |
| R^2^ / R^2^ adjusted | 0.081 / -0.001 |  | 0.027 / -0.060 |  | 0.148 / 0.073 |  | 0.089 / 0.008 |  |

|  | **IIV Intensity variance** |  | **IIV Local shimmer** |  | **IIV Local jitter** |  |
| --- | --- | --- | --- | --- | --- | --- |
| *Predictors* | *Estimates* | *95%CI* | *Estimates* | *95%CI* | *Estimates* | *95%CI* |
| (Intercept) | -0.11 | -38.69 – 38.47 | 0.00 | -0.10 – 0.11 | 0.00 | -0.00 – 0.01 |
| Amyloid status | 2.19 | -4.14 – 8.52 | -0.00 | -0.02 – 0.01 | -0.00 | -0.00 – 0.00 |
| Age | 0.13 | -0.46 – 0.72 | 0.00 | -0.00 – 0.00 | -0.00 | -0.00 – 0.00 |
| Sex | 0.96 | -5.83 – 7.75 | 0.01 | -0.01 – 0.03 | -0.00 | -0.00 – 0.00 |
| Education | 0.46 | -0.48 – 1.39 | 0.00 | -0.00 – 0.00 | 0.00 | -0.00 – 0.00 |
| Observations | 50 |  | 50 |  | 50 |  |
| R^2^ / R^2^ adjusted | 0.051 / -0.033 |  | 0.060 / -0.024 |  | 0.058 / -0.025 |  |

1. **Journaling**

|  | **IIV Long pauses** |  | **IIV Medium pauses** |  | **IIV Pause-to-word ratio** |  | **IIV Pause duration** |  |
| --- | --- | --- | --- | --- | --- | --- | --- | --- |
| *Predictors* | *Estimates* | *95%CI* | *Estimates* | *95%CI* | *Estimates* | *95%CI* | *Estimates* | *95%CI* |
| (Intercept) | -0.01 | -0.06 – 0.03 | -0.08 | -0.20 – 0.03 | **0.18** | **0.03 – 0.33** | 0.20 | -0.37 – 0.76 |
| Amyloid status | 0.00 | -0.01 – 0.01 | -0.01 | -0.03 – 0.00 | -0.01 | -0.03 – 0.02 | 0.00 | -0.09 – 0.09 |
| Age | 0.00 | -0.00 – 0.00 | **0.00** | **0.00 – 0.00** | -0.00 | -0.00 – 0.00 | 0.00 | -0.01 – 0.01 |
| Sex | -0.00 | -0.01 – 0.00 | 0.02 | -0.00 – 0.04 | 0.02 | -0.01 – 0.04 | -0.05 | -0.15 – 0.04 |
| Education | 0.00 | -0.00 – 0.00 | -0.00 | -0.00 – 0.00 | -0.00 | -0.01 – 0.00 | -0.00 | -0.01 – 0.01 |
| Observations | 50 |  | 50 |  | 50 |  | 50 |  |
| R^2^ / R^2^ adjusted | 0.066 / -0.017 |  | 0.156 / 0.081 |  | 0.143 / 0.067 |  | 0.030 / -0.056 |  |

|  | **IIV Phonation rate** |  | **IIV Total audio duration** |  | **IIV Fundamental frequency** |  | **IIV Intensity** |  |
| --- | --- | --- | --- | --- | --- | --- | --- | --- |
| *Predictors* | *Estimates* | *95%CI* | *Estimates* | *95%CI* | *Estimates* | *95%CI* | *Estimates* | *95%CI* |
| (Intercept) | -0.03 | -0.22 – 0.17 | 5.18 | -59.99 – 70.35 | 2.43 | -9.02 – 13.89 | 2.61 | -0.32 – 5.54 |
| Amyloid status | 0.00 | -0.03 – 0.04 | -3.94 | -14.63 – 6.75 | -0.66 | -2.54 – 1.22 | 0.05 | -0.43 – 0.53 |
| Age | 0.00 | -0.00 – 0.00 | 0.37 | -0.63 – 1.37 | 0.01 | -0.16 – 0.19 | -0.01 | -0.06 – 0.03 |
| Sex | -0.00 | -0.03 – 0.03 | -0.37 | -11.84 – 11.10 | **2.75** | **0.74 – 4.77** | -0.15 | -0.67 – 0.36 |
| Education | -0.00 | -0.01 – 0.00 | -0.38 | -1.96 – 1.20 | 0.07 | -0.20 – 0.35 | -0.01 | -0.08 – 0.06 |
| Observations | 50 |  | 50 |  | 50 |  | 50 |  |
| R^2^ / R^2^ adjusted | 0.040 / -0.045 |  | 0.022 / -0.065 |  | 0.160 / 0.085 |  | 0.013 / -0.074 |  |

|  | **IIV Intensity variance** |  | **IIV Local shimmer** |  | **IIV Local jitter** |  |
| --- | --- | --- | --- | --- | --- | --- |
| *Predictors* | *Estimates* | *95%CI* | *Estimates* | *95%CI* | *Estimates* | *95%CI* |
| (Intercept) | 0.54 | -48.96 – 50.03 | 0.04 | -0.08 – 0.17 | -0.00 | -0.00 – 0.00 |
| Amyloid status | -3.43 | -11.56 – 4.69 | -0.01 | -0.03 – 0.01 | 0.00 | -0.00 – 0.00 |
| Age | 0.47 | -0.29 – 1.23 | -0.00 | -0.00 – 0.00 | 0.00 | -0.00 – 0.00 |
| Sex | 2.32 | -6.39 – 11.03 | 0.02 | -0.00 – 0.04 | -0.00 | -0.00 – 0.00 |
| Education | -0.83 | -2.02 – 0.37 | 0.00 | -0.00 – 0.00 | 0.00 | -0.00 – 0.00 |
| Observations | 50 |  | 50 |  | 50 |  |
| R^2^ / R^2^ adjusted | 0.066 / -0.017 |  | 0.092 / 0.012 |  | 0.053 / -0.032 |  |

*Note.* Significant effects are in bold.
